# 16S rRNA Gene Amplicon Sequencing of Gut Microbiota in Gestational Diabetes Mellitus and Type 2 Diabetes Mellitus

**DOI:** 10.1155/jdr/3036209

**Published:** 2026-05-12

**Authors:** Yao Qing, Huanyu Zhou, Chaomeng Zhou, Zhe Song, Jianbo Gao, Jinhua Wei

**Affiliations:** ^1^ Department of Endocrinology, Nanchong Hospital of Beijing Anzhen Hospital Affiliated to Capital Medical University Nanchong Central Hospital, Nanchong, Sichuan, China; ^2^ Department of Gynecology and Obstetrics, Wuxi No. 2 People′s Hospital, Wuxi, Jiangsu, China; ^3^ Department of Obstetrics, Dalian Medical University, Dalian, Liaoning, China, dlmedu.edu.cn; ^4^ Department of Endocrinology and Metabolism, The Affiliated Changzhou No. 2 People′s Hospital of Nanjing Medical University, Changzhou, Jiangsu, China; ^5^ Department of Obstetrics, The Affiliated Changzhou No. 2 People′s Hospital of Nanjing Medical University, Changzhou, Jiangsu, China

**Keywords:** 16S rRNA high-throughput sequencing, gestational diabetes mellitus, gut microbiota, Type 2 diabetes mellitus

## Abstract

**Objective:**

The objective of this study was to investigate the characteristics of the gut microbiota (GM) in pregnant women with gestational diabetes mellitus (GDM) and those with Type 2 diabetes mellitus (T2DM).

**Methods:**

Clinical data from 33 pregnant Chinese women (15 with GDM and 18 without GDM) and 28 nonpregnant Chinese women (14 with T2DM and 14 without T2DM) were collected and statistically analyzed using SPSS software (Version 21.0). Normally distributed data were compared using an independent samples *t*‐test, whereas nonnormally distributed data were compared using the Mann–Whitney *U* test. All statistical tests were two‐sided, and *p* < 0.05 was considered significant. The 16S rRNA gene amplicons of the GM were sequenced using the Illumina HiSeq 2500 platform and compared for differences in genomic diversity, structure, and abundance using bioinformatics.

**Results:**

Significant differences in beta diversity were observed between pregnant women with GDM and healthy pregnant women, as well as between women with T2DM and healthy women. However, pregnant women with GDM and healthy pregnant women did not exhibit significant differences in alpha diversity, whereas women with T2DM and healthy women did. Additionally, the abundance of Firmicutes in pregnant women with GDM was higher than that in healthy pregnant women, whereas the abundances of Bacteroidetes and *Lachnobacterium* were lower (*p* < 0.05). The abundance of *Phascolarctobacterium* in women with T2DM was higher than that in healthy women, whereas the abundances of Proteobacteria, Actinobacteria, *Roseburia*, *Turicibacter*, and *Streptococcus* were lower (*p* < 0.05).

**Conclusion:**

The GM in pregnant women with GDM and those with T2DM differed significantly from that in the control group, providing a reference for future clinical attempts to apply intestinal microecological agents in the treatment of GDM and T2DM.

## 1. Introduction

Recently, the incidence of gestational diabetes mellitus (GDM), which has emerged as a global epidemic, has increased [1, 2]. GDM tends to progress to Type 2 diabetes mellitus (T2DM) after delivery; research suggests that this risk increases approximately sevenfold [3]. GDM is a metabolic disorder characterized by impaired glucose regulation during pregnancy, which can lead to complications such as cesarean or difficult delivery, large‐for‐gestational‐age infants, and low blood sugar levels [4, 5]. T2DM is characterized by insulin resistance and chronic low‐grade inflammation, which affect metabolic homeostasis. These conditions can damage vital organs such as the eyes, kidneys, and heart. Serious risks include hyperosmolar hyperglycemic syndrome and diabetic ketoacidosis [6, 7]. Considering the adverse effects of GDM and T2DM, novel treatments are urgently needed.

The pathogeneses of GDM and T2DM are remarkably similar and primarily involve insulin resistance [8]. Recently, the link between the increasing incidence of both conditions and alterations in the gut microbiota (GM) has become a topic of interest [9–11]. Healthy GMs are crucial for maintaining the intestinal epithelial barrier integrity. Microbial imbalance can lead to a reduction in the expression of tight junction proteins, thereby increasing intestinal permeability and resulting in “leaky gut” syndrome [11]. This allows bacterial endotoxins, such as lipopolysaccharide (LPS), to translocate into the bloodstream, where they activate the Toll‐like Receptor 4 (TLR4) signaling pathway, leading to chronic low‐grade inflammation and insulin resistance. Additionally, the GM converts primary bile acids into secondary bile acids through the action of bile acid hydrolases and hydroxysteroid dehydrogenases. These secondary bile acids act as key signaling molecules that activate the Farnesoid X receptor and the G protein–coupled bile acid Receptor 1, subsequently regulating several downstream pathways involved in glucose homeostasis, insulin secretion, energy expenditure, and inflammatory responses, which are crucial for maintaining metabolic health [12, 13]. Thus, the GM profoundly influences host glucose metabolism and insulin sensitivity through these two mechanisms, which are closely associated with the development and progression of GDM and T2DM.

Several landmark studies have provided evidence of significant alterations in the GM of patients with T2DM and have identified functional changes in the GM associated with T2DM characteristics. Karlsson et al. identified multiple GM alterations in European women with T2DM, including a significantly increased abundance of four *Lactobacillus* species and a significantly decreased abundance of five *Clostridium* species [14]. Qin et al. further identified two butyrate‐producing bacteria in the GM of patients with T2DM, *Roseburia intestinalis* and *Faecalibacterium prausnitzii*, which exhibited high discriminatory power for T2DM. Moreover, functional analysis of the GM revealed increased activity in patients with T2DM related to glucose membrane transport, branched‐chain amino acid transport, and intestinal oxidative stress responses. This study represents significant progress in understanding the correlation between GM alterations and T2DM [15]. Pedersen et al. observed elevated levels of branched‐chain amino acids in patients with insulin resistance [16]. Further mouse experiments revealed that *Prevotella copri* and *Bacteroides vulgatus*, which are enriched in patients with T2DM, increase circulating branched‐chain amino acid levels and induce insulin resistance in mice [17]. These two studies provide crucial complementary insights into the relationship between the GM and T2DM.

Similarly, several studies have revealed the relationship between the GM and GDM. Koren et al. found significant changes in the GM from early to late pregnancy in healthy pregnant women. They transplanted fecal matter from both stages into germ‐free mice. The late‐pregnancy microbiota induced obesity and insulin resistance in mice [18], suggesting that changes in the GM may promote the development of GDM. Subsequently, Kuang et al. and Crusell et al. observed significant alterations in the GM of pregnant women with GDM compared to healthy pregnant women; Kuang et al. reported increased levels of harmful bacteria (such as *Parabacteroides distasonis*, *Klebsiella variicola*, and *Catenibacterium mitsuokai*) and reduced levels of beneficial bacteria (such as *Bifidobacterium* spp., *Eubacterium* spp., and *Alistipes* spp.) in pregnant women with GDM [19]. Crusell et al. identified differences at various taxonomic levels, including the phyla and genera, primarily involving Actinobacteria, *Collinsella*, *Rothia*, and *Desulfovibrio* [20]. Although the results varied, both studies provided strong evidence supporting an association between GM and GDM.

The GM is believed to be associated with the development of GDM and T2DM; however, its specific mechanisms of action remain unclear. Therefore, this study is aimed at investigating the GM of patients with GDM and T2DM, determining the possible causes, and identifying microbiota with large differences. The objective was to lay the foundation for the use of gut microecological agents in the treatment of GDM and T2DM.

## 2. Materials and Methods

### 2.1. Study Groups and Characteristics

Between February 2018 and March 2021, 61 eligible participants visited the Departments of Obstetrics, Endocrinology, or Physical Examination at the Second People′s Hospital of Changzhou, affiliated with Nanjing Medical University. Of these, 15 pregnant women with GDM were enrolled in Group G and met the World Health Organization (WHO)/International Association of Diabetes and Pregnancy Study Group 2013 guidelines [21], whereas 18 healthy pregnant women were enrolled in Group N. Another 14 patients with T2DM were enrolled in Group D, who met the 1999 WHO diagnostic criteria [22], and 14 healthy women were enrolled in Group A. The inclusion criteria for the four groups were as follows: (1) Chinese females aged 20–40 years; (2) no antibiotic treatment within 1 month before sampling and no probiotic preparation within 2 weeks; (3) no irritable bowel syndrome, inflammatory bowel disease, or other gastrointestinal diseases; (4) no history of tumor diseases or organ insufficiencies, such as heart, kidney, or liver disease; and (5) no endocrine diseases, such as thyroid or autoimmune diseases. Written informed consent was obtained from all participants, which was approved by the Ethics Committee of Changzhou Second People′s Hospital, affiliated with Nanjing Medical University (Approval No. 2021 KY011‐01). The study adhered to the principles outlined in the Declaration of Helsinki.

### 2.2. Sample and Data Collection

Fresh stool samples were collected from participants early in the morning. To avoid contamination of surfaces and urine, 3–5 g of feces were collected in sterile, airtight tubes using sterile spoons and immediately stored in a −80°C refrigerator. Patient demographics, including age, weight, height, and blood pressure, were also collected.

### 2.3. Sample DNA Extraction and Gene Sequencing

The QIAamp PowerFecal Pro DNA Kit (QIAGEN, Hilden, Germany) was used to extract total bacterial DNA from the fecal samples. DNA concentration was measured using a Qubit fluorometer, and quality was assessed by running an aliquot on a 1% agarose gel. A PCR system was then configured with a 30‐ng sample of genomic DNA of known mass and corresponding fusion primers, with PCR parameters set for amplification. The V4 region of the bacterial 16S rRNA gene was amplified using degenerate PCR primers 515F (5′‐GTG CCA GCM GCC GCG GTA A‐3′) and 806R (5′‐GGA CTA CHV GGG TWT CTA AT‐3′). The obtained PCR products were subjected to high‐throughput sequencing (using paired‐end sequencing) on an Illumina HiSeq 2500 platform, and sequencing libraries were constructed. Sequencing was performed by BGI Genomics (BGI Research Institute, Wuhan, China).

### 2.4. Bioinformatics Analysis

Raw read data were filtered to remove adapter sequences, low‐quality bases, and uncertain bases. The resulting sequences were assembled into tags and clustered into operational taxonomic units (OTUs) based on overlapping relationships. This step was performed using the USEARCH software (Version 7.0.1090) at a similarity threshold of 97%. Representative OTU sequences were classified using the Ribosomal Database Project Classifier (RDP Classifier, V2.2) with a minimum confidence threshold of 0.6. Species annotation was performed using the Greengenes database (Version 2.01305) in QIIME (Version 1.8.0). Finally, species diversity and intergroup differences were analyzed based on the OTU and species annotation results.

### 2.5. Statistical Analyses

The results were analyzed using SPSS software (Version 21.0). Data that satisfied a normal distribution were represented as the mean ± standard deviation, and group comparisons were performed using the independent samples *t*‐test. Data that did not fit the normal distribution were presented as the median (minimum, maximum), and group comparisons were performed using the Mann–Whitney *U* test. All statistical tests were two‐sided, and *p* < 0.05 was considered significant.

## 3. Results

### 3.1. Clinical Characteristics of Participants

Group G had significantly higher 1‐ and 2‐h oral glucose tolerance test glucose levels than Group N (*p* < 0.001). No discrepancies were observed in gestational week, height, weight, systolic blood pressure, diastolic blood pressure, fasting blood glucose, triglyceride, or total cholesterol levels (*p* > 0.05), except for elevated body mass index (BMI) and age (*p* < 0.05; Table S1a). Furthermore, Group D exhibited considerably higher fasting blood glucose levels (*p* < 0.001). However, no differences in age, height, systolic blood pressure, diastolic blood pressure, triglyceride, or total cholesterol levels were observed when compared with Group A (*p* > 0.05), except for increased weight and BMI (*p* < 0.05; Table S1b).

### 3.2. Identification and Analysis of OTUs Among Groups

**Figure 1:**
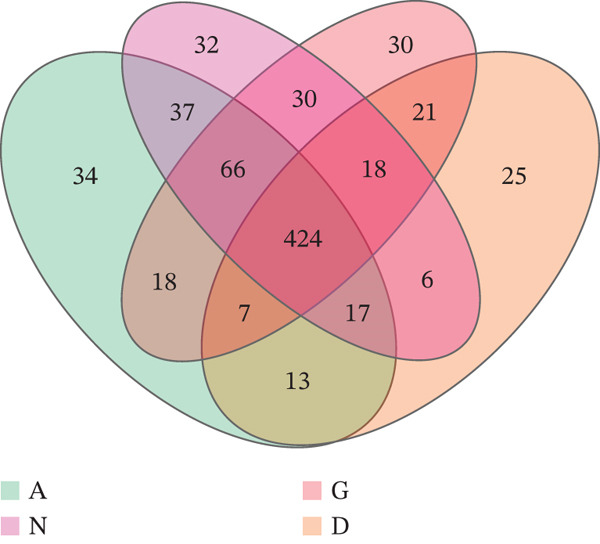
A: Group A (healthy women); N: Group N (healthy pregnant women); G: Group G (pregnant women with GDM); and D: Group D (patients with T2DM). OTU petal diagram. Different colors represent different groups. The overlapping section indicates the number of OTUs shared between the two groups. The nonoverlapping part indicates the number of OTUs that are unique to each group.

A Venn diagram was created based on the distribution of OTU numbers among the four groups. The number of OTUs shared by Groups G and N, as well as Groups D and A, was 538 and 461, respectively, indicating a high level of similarity between the groups. The numbers of OTUs specific to Groups G, N, D, and A were 76, 92, 70, and 155, respectively, indicating differences in species distribution (Figure 1).

### 3.3. Analysis of GM Diversity Between Groups

**Figure 2:**
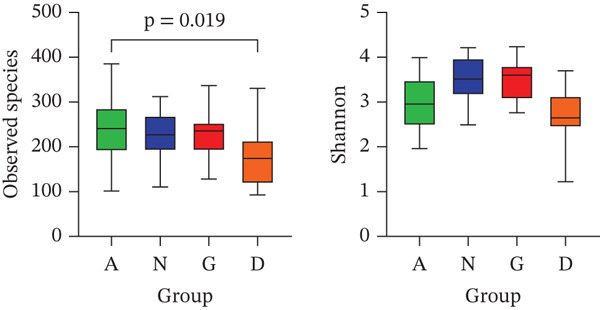
A: Group A (healthy women); N: Group N (healthy pregnant women); G: Group G (pregnant women with GDM); and D: Group D (patients with T2DM). Alpha diversity box plot. The horizontal coordinates indicate the group, and the vertical coordinates indicate the values of the alpha diversity indices, including the observed species index (species richness) and the Shannon index (species diversity). The five lines in the boxes from the bottom to the top represent the minimum, first quartile, median, third quartile, and maximum, respectively. The Wilcoxon rank‐sum test was used for statistical analyses.

The GM diversity analysis was divided into alpha and beta diversity analyses. The alpha diversity of Groups G, N, D, and A was analyzed separately. The observed species index level in Group D was lower than that in Group A (*p* < 0.05), indicating that the species richness in patients with T2DM was lower than that in healthy women. The observed species and Shannon indices in Groups G and N, as well as the Shannon indices in Groups D and A, did not differ significantly (*p* > 0.05). This finding suggests that the GM alpha diversity did not differ between pregnant women with GDM and healthy pregnant women, nor was a difference in species diversity observed between patients with T2DM and healthy women (Figure 2).

**Figure 3:**
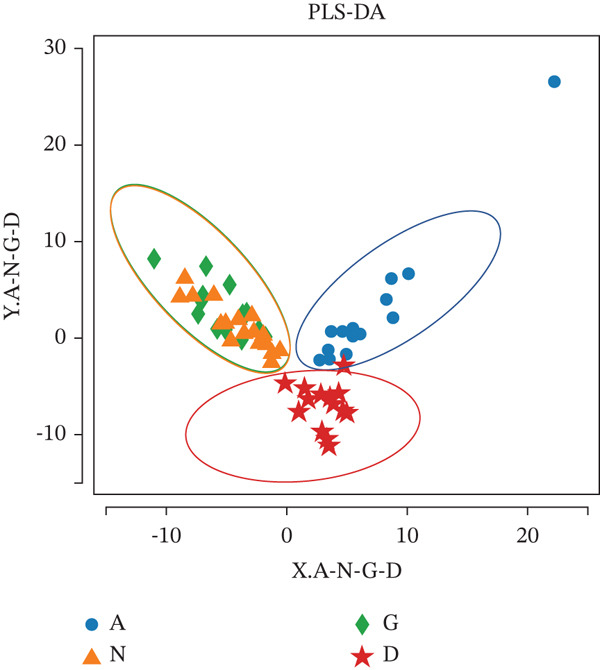
A: Group A (healthy women); N: Group N (healthy pregnant women); G: Group G (pregnant women with GDM); and D: Group D (patients with T2DM). PLS‐DA analysis. The horizontal and vertical coordinates indicate the relative distances, which have no practical significance. A point represents a sample, and a large oval indicates that the GM of each group is differentiated by its respective aggregation.

In contrast, partial least squares discriminant analysis revealed that the GM of Groups G and N collaborated and differed from the GM of Groups D and A. Further analysis of diversity between these groups revealed that the GM of Groups G and N, as well as Groups D and A, were distinct, indicating differences in the way the GM was organized between pregnant women with GDM and healthy pregnant women and between patients with T2DM and healthy women (Figure 3).

### 3.4. Composition and Comparison of GM in Groups

**Figure 4:**
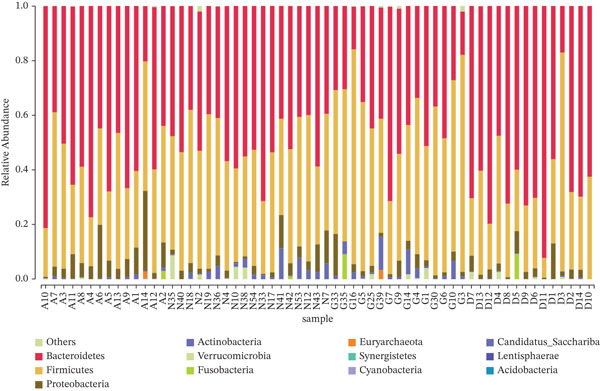
A: Group A (healthy women); N: Group N (healthy pregnant women); G: Group G (pregnant women with GDM); and D: Group D (patients with T2DM). Distribution graph of GM frequency at the phylum level. The horizontal axis shows the sample name, the vertical axis shows the relative abundance of annotated species, and the colors on the right show different species. Unannotated species at each taxonomic level are combined as “unclassified,” and species with < 0.5% abundance in all samples are combined as “others.”

The 12 phyla in the four groups included Firmicutes, Bacteroidetes, Proteobacteria, Actinobacteria, and Verrucomicrobia. The four groups were composed primarily of Bacteroidetes, Firmicutes, and Proteobacteria (Figure 4). In contrast to Group N, the abundance of Firmicutes was significantly increased, whereas that of Bacteroidetes was significantly decreased in Group G (*p* < 0.05; Table S2a). Correspondingly, the abundance of Proteobacteria and Actinobacteria in Group D was significantly lower than that in Group A (*p* < 0.05; Table S2b).

### 3.5. Differential Alteration of GM Across Groups

Linear discriminant analysis effect size revealed that the GM of Groups G and N were similar and distinct from that of Groups D and A (Figure 5a). Furthermore, partial least squares discriminant analysis revealed significant differences in the abundances of Firmicutes, Clostridia, Clostridiales, Bacteroidia, Bacteroidales, *Bacteroides*, and *Lachnobacterium* between Groups G and N (Figure 5b). At the genus level, *Lachnobacterium* abundance was significantly lower in Group G than in Group N (*p* < 0.05). Similarly, the following genera differed significantly between the two groups: Acidaminococcaceae, *Phascolarctobacterium*, Ruminococcaceae, Lachnospiraceae, *Roseburia*, Lactobacillales, and 49 others (Figure 5c). At the genus level, Group D had more *Phascolarctobacterium* and fewer butyrate‐producing bacteria (e.g., *Roseburia* and *Turicibacter*) than Group A. Additionally, Group D had fewer *Streptococcus*, *Actinomyces*, *Coprococcus*, and *Proteus*, as well as many other human commensal bacteria. The differences between the two groups were significant (*p* < 0.05).

Figure 5A: Group A (healthy women); N: Group N (healthy pregnant women); G: Group G (pregnant women with GDM); and D: Group D (patients with T2DM). LEfSe analysis. (a) Five circles representing the taxonomic groups (phylum, class, order, family, and genus) from the innermost to the outermost circle. Different colors indicate different groups. Nodes with the same color as the group indicate the microbial groups that play a crucial role in the group. Each node represents a group of bacteria, and its specific name is shown in the upper‐right corner of the figure. Yellow nodes indicate microbial groups that did not play a crucial role. LDA analysis. (b, c) The groups with LDA scores greater than two (the LDA segmentation threshold). These differences were significant. Different colors represent different groups. The corresponding biological labels played a significant role in these differences. The length indicates the size of a group′s effect on these differences.

**Figure:**
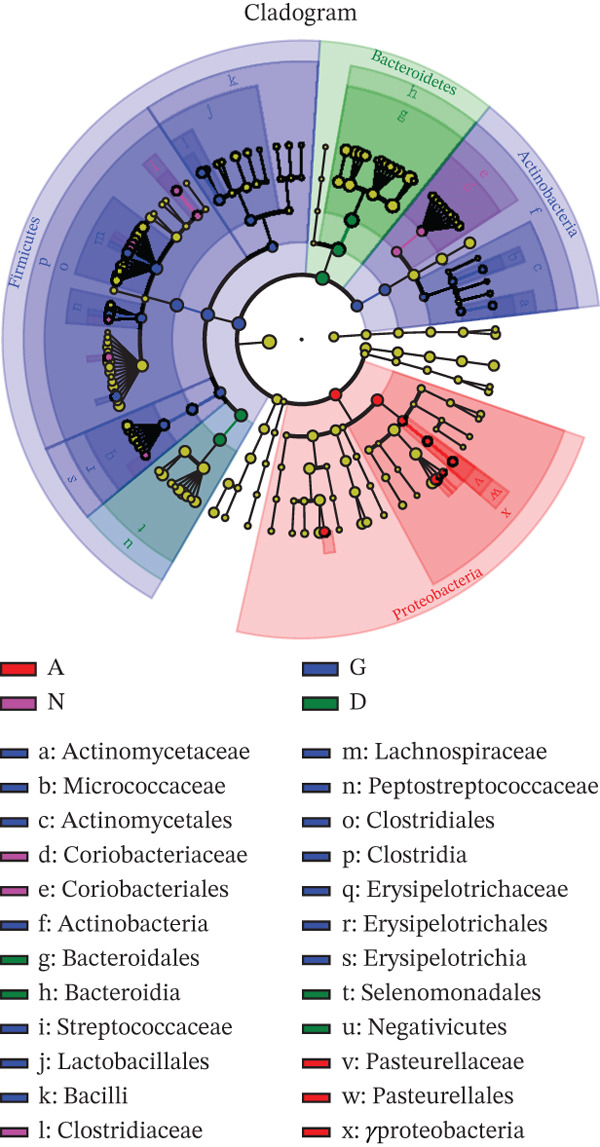
(a)

**Figure:**
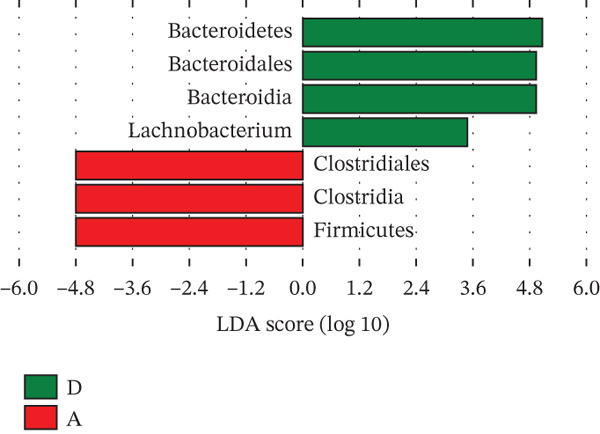
(b)

**Figure:**
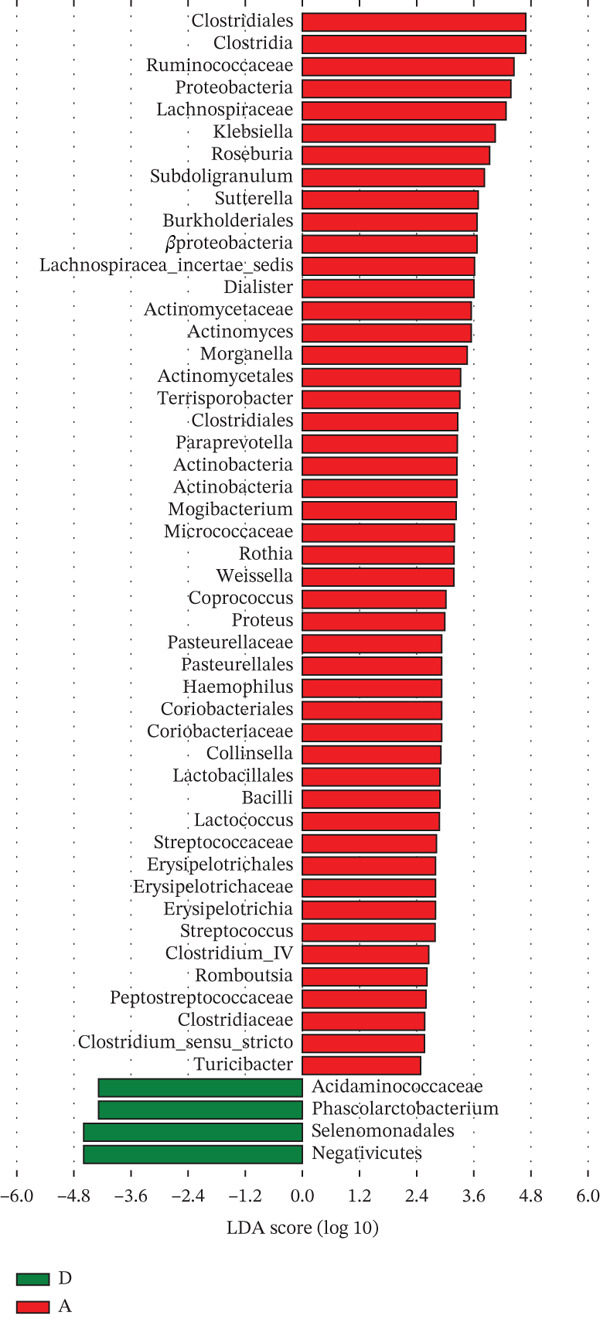
(c)

## 4. Discussion

Major hormonal, immune, and metabolic changes occur during normal pregnancy, with subsequent adaptive changes observed in the maternal GM [23–25]. The GM is more likely to cause obesity and insulin insensitivity in late pregnancy than in early pregnancy [18]. Notably, pregnant women with GDM have reduced insulin sensitivity and significantly dysregulated GM compared to healthy pregnant women [26]. Low‐grade systemic inflammation and insulin resistance are caused by both commensal and pathogenic bacteria [27]. Ferrocino et al. reported that *Blautia* was abundant in pregnant women with GDM. This bacterium was associated with a lower risk of insulin resistance. However, *Bacteroides* was less abundant in pregnant women with GDM. *Bacteroides* was associated with glycated hemoglobin levels [28], suggesting a link between inflammation and metabolic disorders.

The findings of this study suggest a difference in the GM of pregnant women with GDM compared to that of healthy pregnant women at the phylum and genus levels, exhibiting an enriched abundance of Firmicutes and a reduced abundance of Bacteroidetes and *Lachnobacterium*. Firmicutes and Bacteroidetes are the predominant phyla in the human GM, and both contribute to maintaining the body′s energy balance by regulating carbohydrate, fat, and bile acid metabolism [29]. Firmicutes were significantly more abundant in obese populations, whereas Bacteroidetes were less abundant [30]. The increased abundance of Firmicutes showed a significant positive correlation with carbohydrate metabolites such as fructose, galactose, mannitol, and starch, suggesting that it may play a pivotal role in glucose metabolic disorders [27]. Glucose‐related pathways, such as membrane transport and the phosphotransferase system, can transport glucose and catalyze the absorption of carbohydrates through intracellular and outer membranes, stimulating the GM to accelerate glucose conversion into energy [31].

*Lachnobacterium* belongs to the Firmicutes phylum and the Lachnospiraceae family. Various diseases have been linked to a decrease in the number of *Lachnobacterium* in the intestine. For example, a lower number of *Lachnobacterium* during the first 3 months of life was associated with preschool asthma in Canadian children [32]. *Lachnobacterium* has also been associated with the severity of depressive symptoms [33], and this genus was linked to a diminished risk of developing systemic lupus erythematosus [34]. In this study, pregnant women with GDM had significantly lower levels of *Lachnobacterium*, which likely produced fewer short‐chain fatty acids. This finding suggests that harmful bacteria cannot be inhibited and the body produces more substances that cause inflammation. When intestinal inflammatory factors enter the bloodstream, the body produces a constant, mild inflammatory reaction, which can disrupt the structure and function of cells in the pancreas that produce insulin, leading to insufficient insulin secretion and the development of GDM or T2DM [35]. In summary, *Lachnobacterium* is an important GM that requires further exploration in larger cohorts to elucidate the mechanism of action of diabetes.

The number of bacteria in the GM of patients with T2DM was lower than that in healthy women. Additionally, the *β*‐diversity and composition of the GM differed significantly between the two groups at the phylum and genus levels.

Diet is among the most critical environmental factors influencing the structure of the GM [36, 37]. For example, European children who consumed calorie‐rich, fat‐rich, and fiber‐poor diets had higher numbers of Proteobacteria than those in Burkina Faso [38]. Certain dietary components, such as artificial sweeteners and emulsifiers (food additives), impair glucose metabolism in the body and can potentially lead to Proteobacteria blooms [39]. Notably, Proteobacteria were enriched in patients with T2DM. This study found that the abundance of Proteobacteria in patients with T2DM was lower than that in healthy women. This may be due to population heterogeneity, disease stage, or methodological differences. Proteobacteria‐derived LPS can induce metabolic endotoxemia and activate systemic, chronic, and low‐grade inflammation. This represents a key pathway in the pathogenesis of T2DM [40]. The core mechanism pathways are as follows [11]: (i) The “Western diet” promotes the growth of gram‐negative bacteria such as Proteobacteria, leading to an expansion of the LPS‐producing “bacterial reservoir” and compromising intestinal barrier integrity; (ii) LPS translocates through the damaged intestinal mucosa into the bloodstream, causing endotoxemia; (iii) circulating LPS binds to LPS‐binding protein and interacts with receptor complexes (CD14/TLR4/MD2) on immune cell surfaces, thereby activating downstream inflammatory signaling pathways; and (iv) multiple inflammatory mediators such as tumor necrosis factor‐*α*, interleukin‐1*β*, and interleukin‐6 interfere with insulin signaling through various molecular mechanisms, leading to insulin resistance, inducing *β*‐cell dysfunction and apoptosis, and ultimately resulting in T2DM. In summary, alterations in Proteobacteria abundance revealed the complexity of GM dysbiosis in patients with T2DM. Future studies should integrate metagenomics and LPS activity measurements to elucidate the effect of changes in Proteobacteria on systemic inflammation.

The Actinobacteria phylum is another major component of human GM [41]. *Bifidobacterium*, classically beneficial bacteria, are important members of the intestinal flora of humans and animals [42]. The number of *Bifidobacterium* increased significantly after a dietary intervention aimed at reducing body mass in obese children [43]. In addition, the GM of normal and slim mice was transplanted into obese mice, and the abundance of Actinobacteria in the obese mice increased after transplantation with GM from slim mice [44]. In the present study, the abundance of Actinobacteria was reduced in patients with T2DM. Significantly higher total bile acid levels in the plasma were observed in patients with T2DM than in healthy controls, and a decrease in total and secondary bile acid levels and an increase in primary bile acid levels were noted with the use of glucose‐lowering drugs, accompanied by changes in GM, as evidenced by an increase in the abundance of Actinobacteria [45, 46]. Therefore, Actinobacteria may play a role in T2DM by influencing bile acid and glycolipid metabolism.

*Phascolarctobacterium* is typically positively correlated with health status, and its abundance is often reduced in patients with diabetes or in obese individuals [47, 48]. Notably, in this study, an increase in *Phascolarctobacterium* genus abundance was observed in the T2DM cohort. This discrepancy may highlight the context‐dependent nature of GM associations with disease. The following factors may explain this phenomenon: (i) the unique demographic and dietary backgrounds of the current study population, which may shape a distinct microbial ecology; (ii) potential functional heterogeneity at the species or strain level within this genus that cannot be resolved by 16S rRNA sequencing, warranting further metagenomic investigation; and (iii) specific disease status or complications in the enrolled patients. Therefore, the findings of this study highlight the need to validate microbial biomarkers across diverse populations and investigate their functions at a higher resolution to fully understand their roles in the pathogenesis of T2DM.

*Roseburia* and *Turicibacter* are the two primary bacteria that produce butyric acid. The hypothesis that butyric acid promotes the insulin response is supported by an independent clinical study, which is a trial of GM intervention in 35 healthy individuals, showing that reducing the levels of butyrate‐producing bacteria through supplementation with probiotics adversely affected glucose metabolism [46]. This study holds great promise for the therapeutic regulation of blood glucose, such as butyrate production, through GM intervention, as well as for early T2DM treatment and prevention.

Additionally, a healthy GM is characterized by an abundance of bacteria, a complex and stable structure, and a strong resistance to external environmental stresses, such as drugs, foods, and physiological changes in the human body [49]. Patients with T2DM in this study had lower OTU abundance and species richness as well as fewer genera than healthy women, indicating that the GM structure of patients with T2DM was drastically altered. Therefore, treatment of T2DM in the future may be possible by regulating the GM.

GM evolves with age and is primarily characterized by reduced diversity and changes in the core microbiota [50]. Notably, obesity is a potent driver of gut dysbiosis, typically characterized by decreased microbial diversity, altered Firmicutes/Bacteroidetes ratios, and increased proinflammatory bacteria [51]. This “age‐ or obesity‐associated microbiota” may substantially overlap with the microbial profiles observed in T2DM and GDM [11]. Wu et al. adjusted for multiple covariates, including BMI and age, to analyze the association between the GM and T2DM. The association between certain microbiota and T2DM weakened or even disappeared [52], highlighting the importance of age and BMI as confounding factors. Crusell et al. noted that the GM of women with GDM differs from that of healthy pregnant women. They specifically highlighted that the prepregnancy BMI is a major factor influencing the composition of the microbiota. After adjustment, the GM of women with GDM exhibited distinct patterns, yet specific microbial biomarkers persisted (e.g., enrichment of the *Collinsella* genus) [20]. This finding suggests that some microbial alterations occur independent of BMI, whereas others are BMI‐dependent. In summary, age and BMI must be controlled for as confounding factors when conducting studies on the association between GM and GDM or T2DM. However, the current study did not control for age and BMI, and these variables were not sufficiently adjusted in the model, which may have introduced potential confounding effects on the GM association results. Therefore, future studies with larger sample sizes and matched designs will be conducted to validate the findings.

This study has several advantages. First, the study population was divided into four groups: GDM, healthy pregnant women, T2DM, and healthy women, allowing comprehensive and convincing data analysis. Second, the study population tended to be young (20–40 years old), which leaves room for guidance in preventing the development of GDM into T2DM in terms of disease plasticity. However, this study had some limitations. First, the sample size was relatively small; however, it can be expanded later to confirm the results more definitively. Second, age and weight were not fully controlled for, as age and obesity are independent risk factors for GDM and T2DM [37], which are more challenging to control when samples are collected. Future studies should recruit a larger number of participants. Although the study lacked dietary data, all participants were long‐term residents of Changzhou City, Jiangsu Province, China. Data collection was conducted at the Changzhou Second People′s Hospital, affiliated with Nanjing Medical University, with participants matched for baseline characteristics such as age and sex that may influence dietary patterns. Although this cannot entirely rule out dietary differences, it helps to mitigate potential systematic biases. Future studies should incorporate detailed dietary assessments to validate and expand the preliminary findings. Finally, this study employed 16S rRNA gene sequencing, which makes it challenging to detect differences below the genus level of GM. Macroeconomic analysis or other more accurate assays can be used to conduct further studies.

## 5. Conclusion

Pregnant women with GDM had more Firmicutes, and fewer Bacteroidetes and *Lachnobacterium* were noted compared to healthy individuals. Significant changes in the numbers of other bacteria were also noted in patients with T2DM. If these findings are validated in future studies with larger sample sizes, they will provide new avenues for the application of intestinal microecological agents in the treatment of GDM and T2DM.

## Author Contributions

Yao Qing′s and Huanyu Zhou′s contributions to this work were equal.

## Funding

Jiangsu Provincial Maternal and Child Health Association (FYX201807), Changzhou Health Bureau (ZD201811), and Changzhou Science and Technology Bureau (CZ20210025 and MH202508).

## Disclosure

The funders had no role in the study design, data collection and analysis, decision to publish, or preparation of the manuscript.

## Ethics Statement

This study was approved by the Ethics Committee of the Second People′s Hospital of Changzhou, affiliated with Nanjing Medical University, and adhered to the principles of the Declaration of Helsinki. The research protocol was approved under the following number: [2021] KY011‐01. All the participants provided informed consent. The registry was approved, and the study/trial registration number was not applicable (N/A).

## Conflicts of Interest

The authors declare no conflicts of interest.

## Author Biographies

**Jinhua Wei** is currently an associate professor at Changzhou Second People′s Hospital. She underwent a year of training in the Department of Medicine at Yale University. Her research focuses on microbes in the human gut and methods for identifying and diagnosing diabetes, as well as gestational diabetes.

**Jianbo Gao** earned his Ph.D. from Nanjing Medical University. He is currently the chief physician in the endocrinology department at the Second People′s Hospital of Changzhou and an associate professor and master′s supervisor at the Third Affiliated Hospital of Nanjing Medical University. His research interests include diabetes, gestational diabetes mellitus, insulin sensitivity, and insulin resistance.

**Yao Qing** currently works at Nanchong Central Hospital as a hospital doctor. Her research interests include the gut microbiota, diabetes, and gestational diabetes mellitus.

**Huanyu Zhou** currently works at Wuxi No. 2 People′s Hospital as a hospital doctor. Her research interests include the gut microbiota, gestational diabetes mellitus, and lactation.

**Chaomeng Zhou** currently works at Shenzhen Luohu Maternity and Child Healthcare Hospital as a hospital doctor. Her research interests include the gut microbiota, gestational diabetes mellitus, and lactation.

**Zhe Song** currently works at Jiangyin Maternal and Child Health Hospital as a hospital doctor. Her research interests include gestational diabetes mellitus and lactation.

## Supporting information


**????** Additional supporting information can be found online in the Supporting Information section. Table S1: (a) Comparison of clinical data between Groups G and N. (b) Comparison of clinical data between Groups D and A. Table S2: (a) Analysis of GM differences between Groups G and N at the phylum level. (b) Analysis of GM differences between Groups D and A at the phylum level.

## Data Availability

The data that support the findings of this study are available on request from the corresponding authors. The data are not publicly available due to privacy or ethical restrictions.
