# Supplementary material for: 16S rRNA Gene Amplicon Sequencing of Gut Microbiota in Gestational Diabetes Mellitus and Type 2 Diabetes Mellitus
Source: J Diabetes Res. 2026 May 12;2026:3036209. doi: 10.1155/jdr/3036209 (PMC13162232; doi:10.1155/jdr/3036209)
Supplement: Supplementary file 1 — ???? Additional supporting information can be found online in the Supporting Information section. Table S1: (a) Comparison of clinical data between Groups G and N. (b) Comparison of clinical data between Groups D and A. Table S2: (a) Analysis of GM differences between Groups G and N at the phylum level. (b) Analysis of GM differences between Groups D and A at the phylum level. [file JDR-2026-3036209-s001.docx]

Table S1a

Table S1a Comparison of clinical data between group G and group N

| clinical data | Group G | Group N | *P* value |
| --- | --- | --- | --- |
| Number | 15 | 18 | - |
| Age,year | 30.07±3.47 | 26.06±3.57 | ＜0.01 |
| Height,cm | 160.00（150，172） | 160.00（157，172） | 0.33 |
| Weight,kg | 70.43±12.60 | 65.29±7.48 | 0.16 |
| BMI,(kg/m^2^) | 27.21±4.08 | 24.74±2.61 | 0.04 |
| SBP,mmHg | 115.27±9.26 | 115.67±10.18 | 0.91 |
| DBP，mmHg | 68.13±8.90 | 66.28±7.28 | 0.52 |
| Triglycerides,mmol/L | 2.88±0.98 | 2.64±0.89 | 0.48 |
| Total cholesterol,mmol/L | 6.20±1.09 | 5.88±1.08 | 0.40 |
| FBG,mmol/L | 4.53（4.09，6.07） | 4.41（3.98，5.08） | 0.21 |
| 1h OGTT glucose,mmol/L | 10.06±1.54 | 7.11±1.20 | ＜0.001 |
| 2h OGTT glucose,mmol/L | 9.03±1.53 | 6.27±0.87 | ＜0.001 |
| Gestational weeks | 26.42±1.25 | 25.94±1.65 | 0.36 |

Table S1b

Table S1b Comparison of clinical data between group D and group A

| clinical data | Group D | Group A | *P* value |
| --- | --- | --- | --- |
| Number | 14 | 14 |  |
| Age,year | 32.71±4.03 | 29.93±4.71 | 0.11 |
| Height,cm | 160.29±3.29 | 162.71±3.32 | 0.06 |
| Weight,kg | 61.29±7.34 | 55.96±5.21 | 0.04 |
| BMI(kg/m^2^) | 23.79±2.63 | 21.16±2.13 | ＜0.01 |
| SBP,mmHg | 119.71±8.39 | 113.93±6.99 | 0.06 |
| DBP，mmHg | 73.93±6.04 | 72.21±5.03 | 0.42 |
| Triglycerides,mmol/L | 1.76±0.87 | 1.21±0.61 | 0.06 |
| Total cholesterol,mmol/L | 4.77±0.82 | 4.82±0.96 | 0.88 |
| FBG,mmol/L | 9.90（5.06，19.40） | 4.67（4.05，5.69） | ＜0.001 |

Table S2a

Table S2a Analysis of GM differences between groups G and group N at phylum level

|  | Group G | Group N | *P* value |
| --- | --- | --- | --- |
| Firmicutes | 53.03±14.48 | 42.05±8.48 | 0.02 |
| Bacteroidetes | 38.97±14.60 | 49.96±9.35 | 0.01 |

Table S2b

Table S2b Analysis of GM differences between groups D and group A at phylum level

|  | Group D | Group A | *P* value |
| --- | --- | --- | --- |
| Proteobacteria | 3.56±3.46 | 8.08±7.65 | 0.02 |
| Actinobacteria | 0.17±0.16 | 0.52±0.50 | 0.02 |
